# Increasing incidence of pertussis in Brazil: a retrospective study using surveillance data

**DOI:** 10.1186/s12879-015-1222-3

**Published:** 2015-10-23

**Authors:** Lucas Melo Guimarães, Eduilson Lívio Neves da Costa Carneiro, Filipe Anibal Carvalho-Costa

**Affiliations:** Regional Office Oswaldo Cruz Foundation (Fiocruz), Teresina, Piauí Brazil; Laboratory of Epidemiology and Molecular Systematics, Oswaldo Cruz Institute, Fiocruz, Rio de Janeiro Brazil; Federal Institute of Education, Science and Technology of Piauí (IFPI), Teresina, Piauí Brazil

**Keywords:** Pertussis, *Bordetella pertussis*, Epidemiology, Vaccination, Brazil

## Abstract

**Background:**

Many countries have reported an increase in the incidence of pertussis, which has become a global public health concern.

**Methods:**

In this study, the epidemiology of pertussis in Brazil was assessed retrospectively using surveillance data gathered from case notification forms from 2007 to 2014.

**Results:**

From 2007 to 2014, 80,068 suspected cases of pertussis were reported in Brazil. Of these, 24,612 (32 %) were confirmed by various criteria. The annual distribution of confirmed cases demonstrated a significant increase in incidence rate since 2012. A seasonal pattern in which cases occur most frequently between the end of spring and midsummer has been identified. Among the confirmed cases, 34.5 % occurred in infants aged 0–2 months, 22.4 % occurred in infants aged 3–6 months, 21 % occurred in children aged 7 months to 4 years, and 8 % were reported in adults >21 years. Of the confirmed cases, 47.2 % met only clinical criteria, 15.5 % met clinical and epidemiological criteria, and 36.6 % were confirmed in a laboratory. The overall case fatality rate was 2.1 %, reaching 4.7 % among infants aged 0–2 months. The complications most commonly reported in the notification forms were pneumonia, encephalitis, dehydration, otitis, and malnutrition. Of the confirmed cases, 23.1 % occurred in subjects who received at least 3 doses of the pertussis vaccine. Within this group, there were 1098 infants aged 7 to 15 months and 2079 children aged 16 months to 4 years. In 2012, 18 states did not achieve 95 % immunization coverage, a number that dropped to 10 and 6 in 2013 and 2014, respectively.

**Conclusions:**

Brazil’s main challenges in facing pertussis resurgence will be to offer the best quality medical attention to reduce mortality, to improve the infrastructure for laboratory diagnosis and to increase vaccination coverage. Additional studies to assess the effectiveness of the current vaccination schedule and basic research on the genetics and evolution of circulating B. pertussis strains are also needed.

## Background

An increasing incidence of pertussis (whooping cough) has been reported in many countries and currently represents a global public health concern [1, 2]. The disease is a potentially lethal, highly contagious respiratory tract infection caused by the gram-negative bacteria *Bordetella pertussis. B. bronchiseptica*, *B. parapertussis*, and *B. holmesii* can also cause the disease in humans [3, 4]. A pertussis-like syndrome can be caused by respiratory viruses, mycoplasmas, and other bacteria, such as *Haemophilus influenzae* and *Streptococcus pneumoniae* [5].

Pertussis has an incubation period ranging from 5 to 21 days. *B. pertussis* infects the respiratory epithelium of trachea, bronchi and bronchioles, causing an accumulation of mucus and debris in the airways [6]. Clinically, the disease is characterized by a paroxysmal cough and fever and is frequently complicated by episodes of apnoea and cyanosis [7]. The majority of fatal cases have are among patients who develop bronchopneumonia caused by *B. pertussis* or a co-infection with other bacteria [6].

Pertussis is a vaccine-preventable disease. The incidence has been reduced drastically in all countries that have achieved satisfactory levels of vaccine coverage. Pertussis vaccines are combined with diphtheria and tetanus toxoids to produce the diphtheria-tetanus-pertussis (DTP) trivalent vaccine. More recently, adding the hepatitis B virus, *H. influenzae* type B antigens, and/or inactivated poliovirus has led to the development of tetravalent and pentavalent vaccines [8].

The first generation of vaccines, still widely used in developing countries, contains heat-killed whole *B. pertussis* cells (wP) obtained from cultures. These vaccines are highly immunogenic and effective, although they are associated with potentially severe side effects such as febrile seizures [9]. Acellular (aP) vaccines are used in developed countries and are significantly less reactogenic, containing distinct combinations of three to five of the following antigens: pertussis toxin, filamentous hemagglutinin, pertactin, fimbrial antigen 2, and fimbrial antigen 3 [10–12].

In 2002, the Brazilian National Immunization Program (NIP) replaced the first three doses of the diphtheria-tetanus-whole pertussis (DTwP) vaccine with the tetravalent vaccine DTwP + *H. influenzae* type B (DTwP-Hib). In 2012, this vaccine was replaced by the pentavalent vaccine DTwP + *H. influenzae* type B + hepatitis B (DTwP-Hib-HBV). Currently in Brazil, pertussis immunization is with 3 doses of the DTwP-Hib-HBV vaccine given at 2, 4 and 6 months of age, followed by two boosters with DTwP at 15 and 48 months of age. The diphtheria-tetanus-acellular pertussis (DTaP) is given only to infants who have had severe reactions to the DTwP or in private vaccine clinics, and 2014, the NIP began providing it to pregnant women.

Several hypotheses have been proposed to explain the resurgence of the disease, including a decrease in the immunogenicity of aP vaccines, which would lead to early weaning of immunity and result in *B. pertussis* circulating among adolescents and adults and spreading from them to young infants who are not fully immunized. It has also been proposed that *B. pertussis* may have undergone a genetic evolution associated with the selective pressure of vaccines. A significant increase in the pertussis incidence rate has been observed in Brazil in recent years [13]. This study aims to describe aspects of the pertussis resurgence in Brazil, including the spatiotemporal distribution, case fatality rates, confirmation criteria and patient vaccination status.

## Methods

The epidemiology of pertussis in Brazil was studied using surveillance data that were retrospectively gathered from case notification forms from 2007 to 2014. Data that are generally not freely accessible were made available for this study by the Brazilian Ministry of Health. Brazil has a population of 202,768,562 inhabitants spread over 8,515,767 km^2^ and is divided into five geographical regions: the North (which corresponds to the Amazon region with 16,983,485 inhabitants), the Northeast (53,081,510 inhabitants), the Midwest (14,993,194 inhabitants), the Southeast (84,465,579 inhabitants) and the South (27,465,289 inhabitants). Brazil has 26 federative units (states) and one Federal District.

Information concerning the vaccination coverage rates were collected from the website of the Department of Informatics of the Unified Health System (Datasus) [14]. Datasus calculates the immunization coverage rates as the number of children with complete basic scheme in the target age for a particular type of vaccine/number of children in the target age X 100.

Pertussis is a reportable disease in Brazil. All suspected cases treated at the Unified Health System are reported using standardized forms sent to the Information System for Notifiable Diseases (SINAN) of the Ministry of Health. Pertussis notification forms have many variables, including demographic and clinical data, treatment, confirmation criteria, control measures adopted, pertussis vaccination status, hospital discharge and death. This study was conducted with the data available in pertussis reporting forms sent to the Ministry of Health of Brazil from 2007 to 2014.

In Brazil, suspected pertussis cases are confirmed or discarded at the health unit on the basis of clinical, epidemiological or laboratory criteria. A case is confirmed with clinical criteria if the blood count presents 20,000 or more leukocytes/μL and 10,000 or more lymphocytes/μL, with a negative or absent culture, lack of epidemiological link, or without confirmation of aetiology. Epidemiological criteria are met with a confirmed case of pertussis via laboratory testing, which is performed between the beginnings of the catarrhal period until three weeks after the beginning of the disease paroxysmal period. Laboratory criteria are based on the isolation of *B. pertussis* using a culture of nasopharyngeal secretion or positive polymerase chain reaction (PCR). In a variable proportion of patients, a sample of nasopharyngeal secretion is collected and then forwarded to the Central Laboratories of Public Health (LACENs). Confirmation rates were calculated as the number of confirmed cases (by any criteria)/number of notified (suspected) cases.

Population data from the Brazilian Institute of Geography and Statistics, available at www.ibge.gov.br, were used for calculation of incidence rates as the number of pertussis confirmed (by any criteria) cases/population in a defined municipality-state-whole country in a specific year X 100,000 inhabitants. The spatio-temporal and age distribution of cases was plotted and maps were produced with Quantum Geographic Information System (QGIS) software (QGIS Development Team, available at http://www.qgis.org/en/site/). Case fatality rates in different regions, years, and age groups were calculated as the number of deaths/number of confirmed cases X 100. Complications that led to fatalities were assessed. We also examined the vaccination status of notified cases, observing the utilization of DTwP-Hib (tetravalent) vaccine until May 2012, followed by the implementation of DTwP-Hib-HBV (pentavalent) vaccine in June 2012. Children were considered vaccinated if they had received three or more doses of DTwP-Hib or DTwP-Hib-HBV. The proportion of vaccinated subjects among confirmed and discarded cases was compared through the chi-square test with a statistical significance of 5 %. Analyses were performed with EpiInfo 7.1.2.0 (Centers for Disease Control and Prevention, Atlanta, Georgia, USA).

Descriptive statistics are presented, with the distribution of cases by month, year and state, as well as the frequencies of distinct diagnostic criteria used to confirm the cases. The mean incidence rates of pertussis were calculated and presented. The frequencies of clinical complications in the cured group and in the group that evolved to death were compared with chi-square test. The confirmation rates in vaccinated and unvaccinated groups in distinct age strata were also compared using the chi-square test. Statistical significance was established at *p* < 0.05.

The study was approved by the Research Ethics Committee of the Oswaldo Cruz Institute/Fiocruz, license number 39406914.0.0000.5248.

## Results

### Confirmed cases, incidence rates, spatio-temporal and age distribution of pertussis in Brazil

**Fig. 1:**
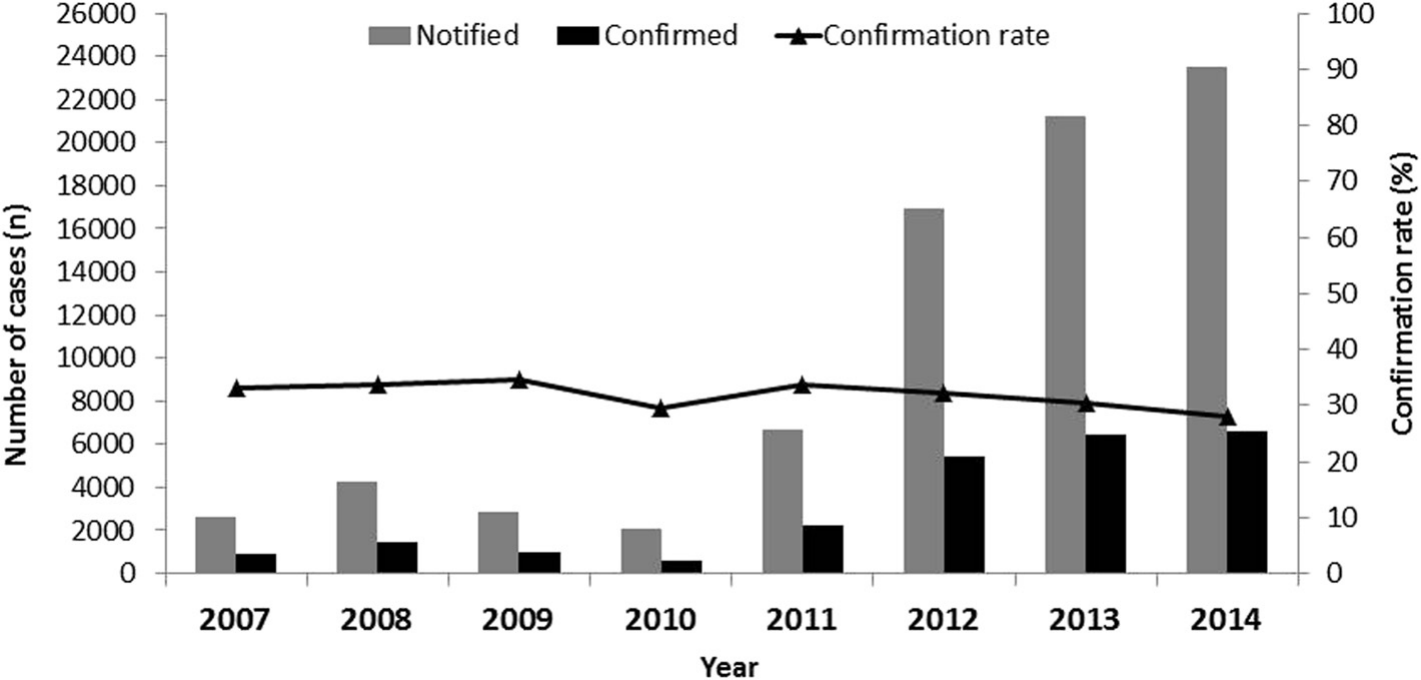
Absolute numbers of reported and confirmed cases of pertussis and confirmation rates (confirmed cases/reported cases X 100) in Brazil, from 2007 to 2014 per year

**Fig. 2:**
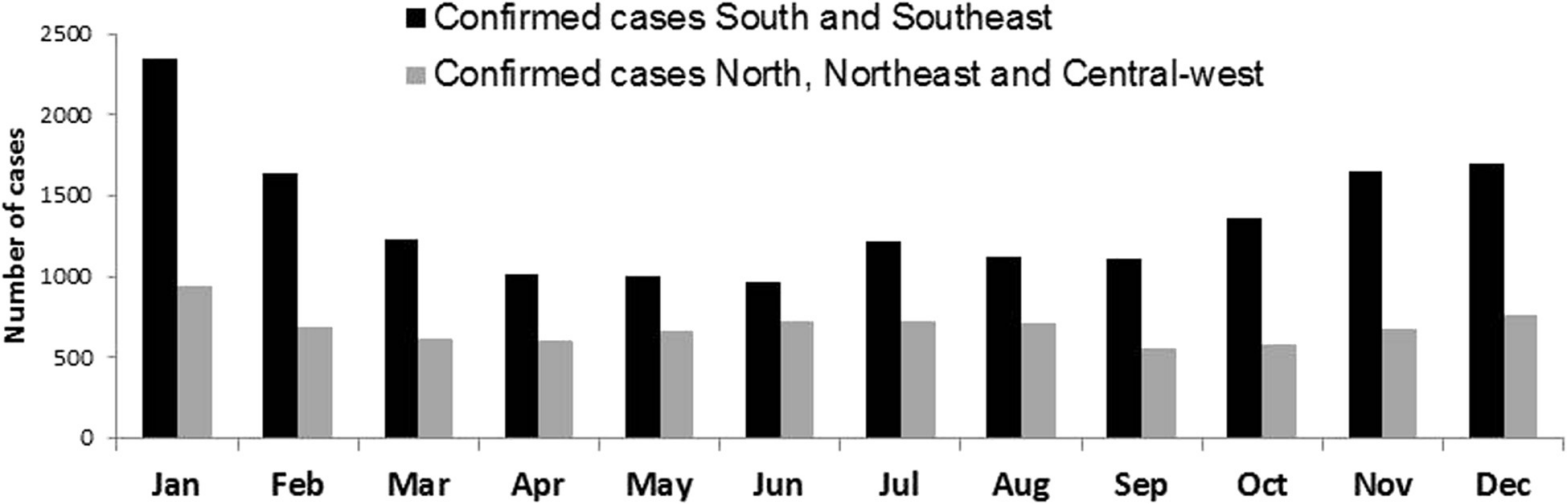
Seasonality of pertussis in Brazil: Number of reported and confirmed cases in different regions of Brazil, by month, 2007–2014. The monthly values represent the sum of confirmed cases between 2007 and 2014 in that particular month

Between January 2007 and December 2014, 80,068 suspected cases of pertussis were reported in Brazil. Of these, 24,612 (32 %) were confirmed using clinical, epidemiological or laboratory criteria. The annual distribution of confirmed cases demonstrated a significant increase in the number of cases from 2012. The yearly average of confirmed cases was 1226 per year between 2007 and 2011, reaching 6161 per year between 2012 and 2014. The confirmation rate was not related to the increase in the number of cases being registered; the highest rate of confirmation was in 2009 (34.7 %), and the lowest was in 2010 (29.6 %). The annual distribution of reported, and the confirmed cases and confirmation rates are depicted in Fig. 1. The distribution of cases by month showed an increase in the period from November to February, as shown in Fig. 2. Therefore, a seasonal pattern, in which cases occur most frequently between the end of spring and midsummer in Southern Hemisphere, has been identified. This seasonality was observed in all five Brazilian regions, but seems to be more marked in the South and Southeast. The annual incidence rates between 2007 and 2010 ranged from 0.32 cases per 100,000 to 0.75 per 100,000, showing an increase in 2011 and reaching 1.17 per 100,000, and then rising to 2.81 per 100,000 in 2012, 3.2 per 100,000 in 2013, and 3.25 per 100,000 in 2014 (not shown). During the epidemic period, the mean annual incidence rate was 2.6 cases per 100,000, compared with 0.51 per 100,000 in the period from 2007 through 2010.

**Fig. 3:**
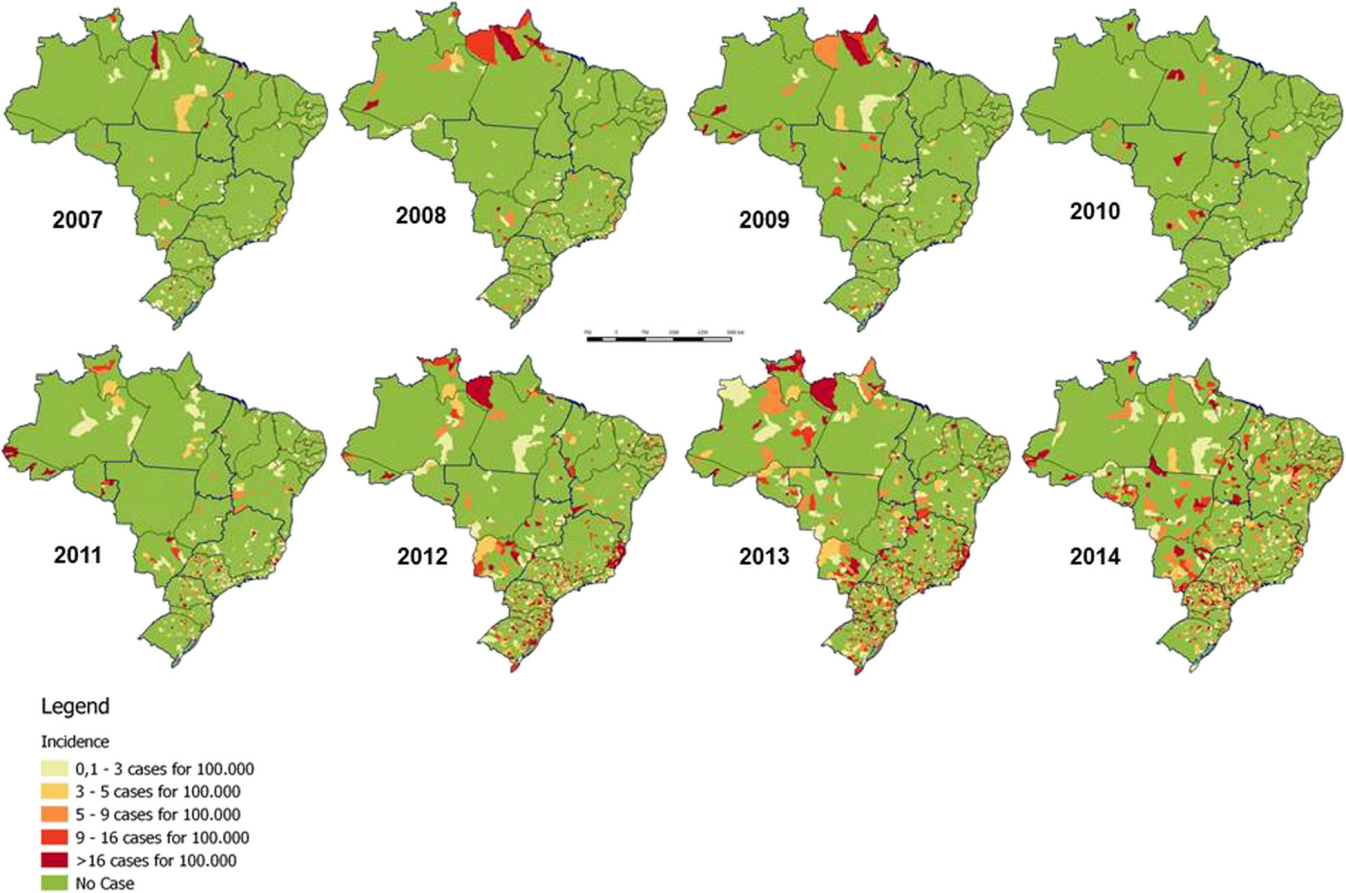
Maps showing distinct strata of pertussis incidence rates in the different Brazilian municipalities, from 2007 to 2014. The maps were made with the QGIS program using study data

With respect to the distribution of cases by state, the states of São Paulo, Espírito Santo, Rio Grande do Sul and Paraná accounted for 53.7 % (*n* = 13,216) of the confirmed cases of pertussis. During the epidemic period, when there were 19,522 confirmed cases, the Southeast region recorded 47.7 % (*n* = 9311), followed by the South with 20.5 % (*n* = 4000) and the Northeast with 20.4 % (*n* = 3988) (data not shown). The annual evolution of pertussis incidence rates from 2007 to 2014 in the different Brazilian municipalities is shown in the maps in Fig. 3. An increased incidence in many municipalities can be observed in 2012.

**Fig. 4:**
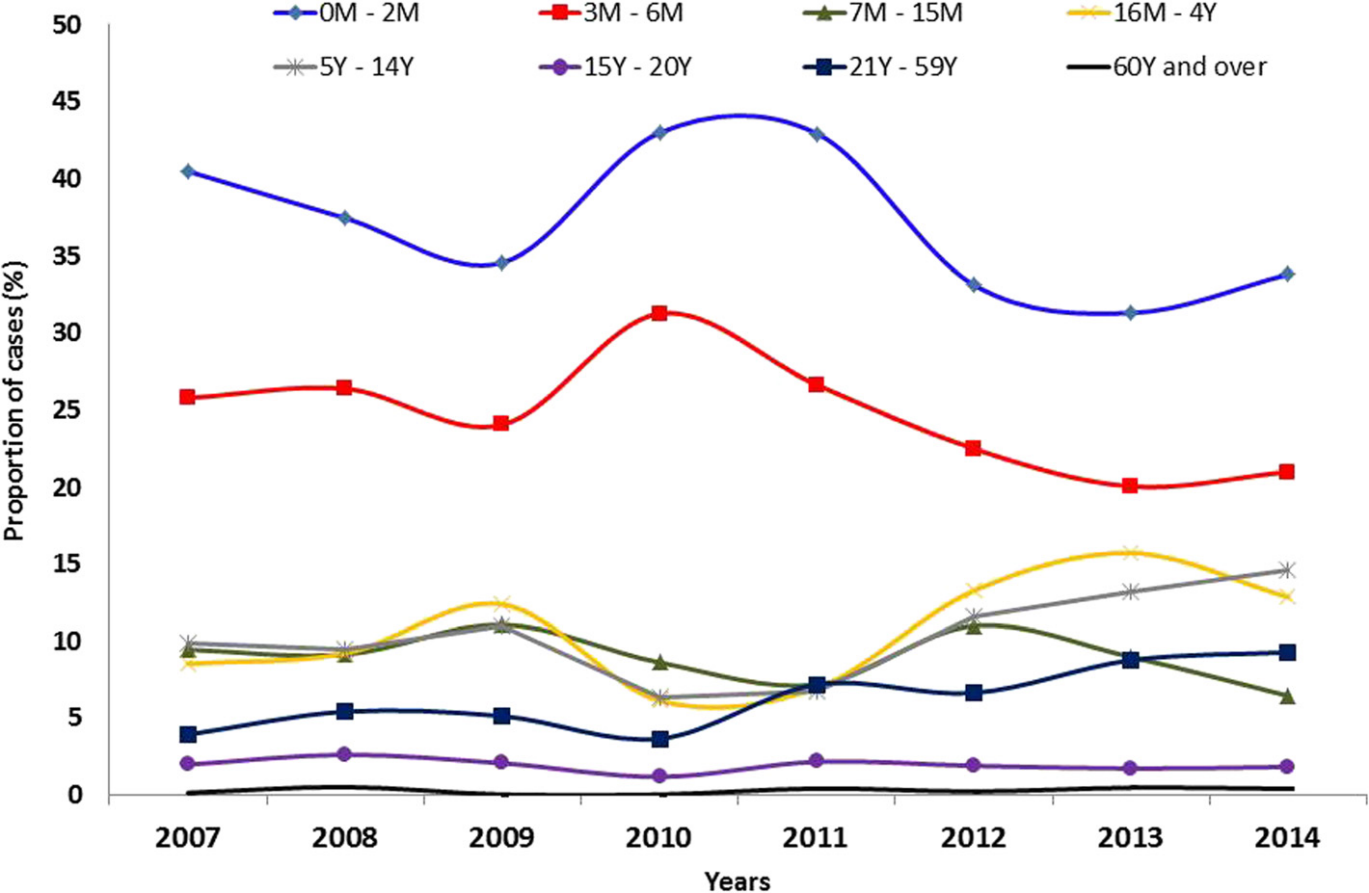
Distribution of confirmed cases of pertussis by age group from 2007 to 2014. The values represent the proportion of cases registered in each age group per year

By studying the distribution of cases by age, we aimed to identify the proportion of cases in age groups in which immunization from vaccines is not expected. These groups included infants aged 0 to 2 months and adults over the age of 21 years. The distribution is shown in distinct years in Fig. 4. Among the confirmed cases, 34.5 % (*n* = 8491) occurred in infants aged 0 to 2 months, and approximately 8 % (*n* = 1969) were reported in adults older than 21 years of age. In observing other age groups, it was noted that 22.4 % (*n* = 5513) were children aged 3 to 6 months, i.e., in the process of immunization, while 21 % (*n* = 5168) occurred in children aged 7 months to 4 years old, who are expected to be immunized. It is observed that in the period 2012–2014, the proportion of cases in children aged 0 to 6 months decreased, while the proportion of the reported pertussis cases among children aged 16 months to 4 years and 5 to 14 years increased. Among the confirmed cases, 55.4 % (*n* = 13,626) occurred in females, 0.5 % (*n* = 73) of whom were pregnant.

### Confirmation criteria for pertussis cases in Brazil

**Fig. 5:**
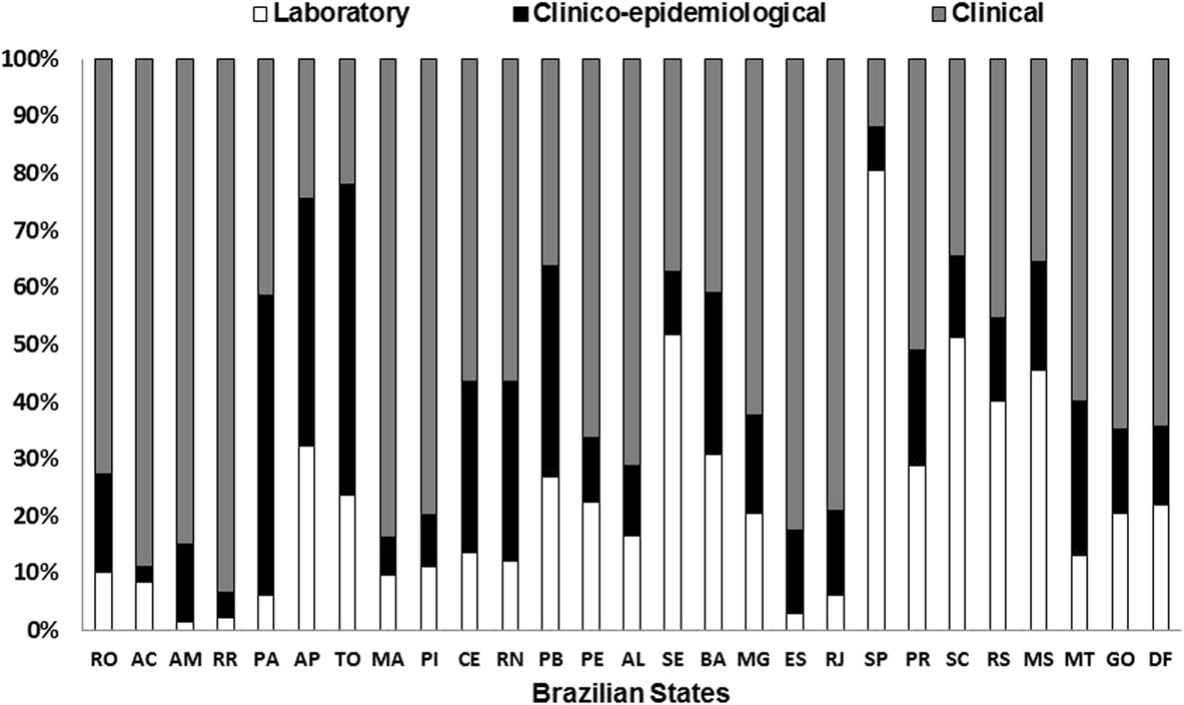
Distribution of the criteria used to confirm the diagnosis of pertussis in Brazil, 2007–2014. The values represent the proportion of cases that were confirmed by each criteria, by state. RO = Roraima, AC = Acre, AM = Amazonas, RR = Roraima, PA = Pará, AP = Amapá, TO = Tocantins, MA = Maranhão, PI = Piauí, CE = Ceará, RN = Rio Grande do Norte, PB = Paraíba, PE = Pernambuco, AL = Alagoas, SE = Sergipe, BA = Bahia, MG = Minas Gerais, ES = Espírito Santo, RJ = Rio de Janeiro, SP = São Paulo, PR = Paraná, SC = Santa Catarina, RS = Rio Grande do Sul, MS = Mato Grosso do Sul, MT = Mato Grosso, GO = Goiás, DF = Distrito Federal (Federal District)

It was observed that 47.2 % (*n* = 11,606) of the confirmed cases met only the clinical criteria, 15.5 % (*n* = 3821) met clinical and epidemiological criteria, and 36.6 % (*n* = 9017) were laboratory confirmed by various methodologies, most frequently the culture for *B. pertussis* of nasopharyngeal secretions. Among the confirmed cases, the laboratory confirmation rate ranged from 24.9 % (2009) to 49.5 % (2011). Important regional differences were also observed; São Paulo showed the highest rates, reaching 80.2 % of laboratory confirmations over the period (Fig. 5).

### Complications and case fatality rates

**Fig. 6:**
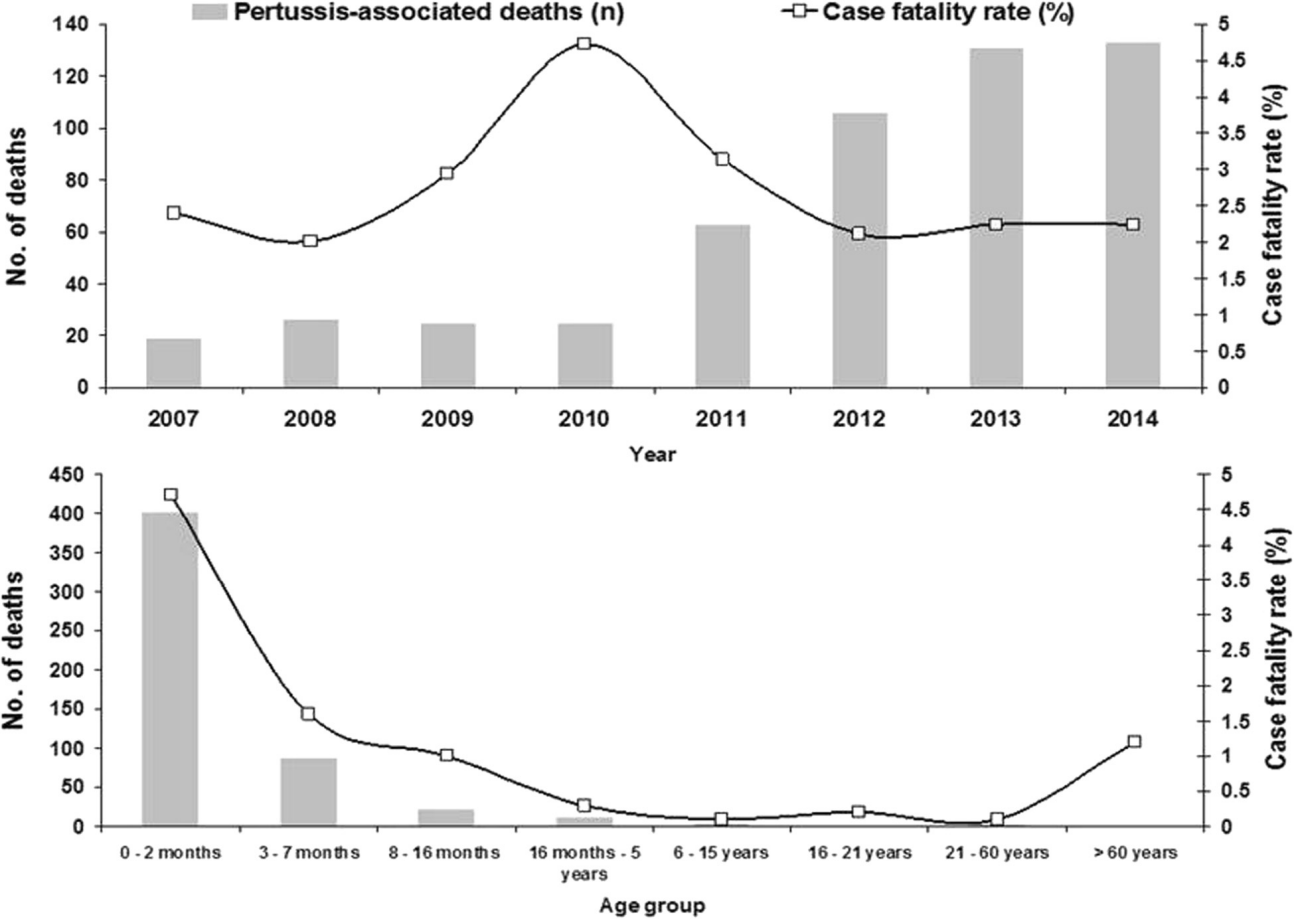
Number of deaths and pertussis case fatality rates in Brazil, from 2007 to 2014 in different age groups. Case fatality rates were calculated as the number of deaths/number of confirmed cases X 100

**Fig. 7:**
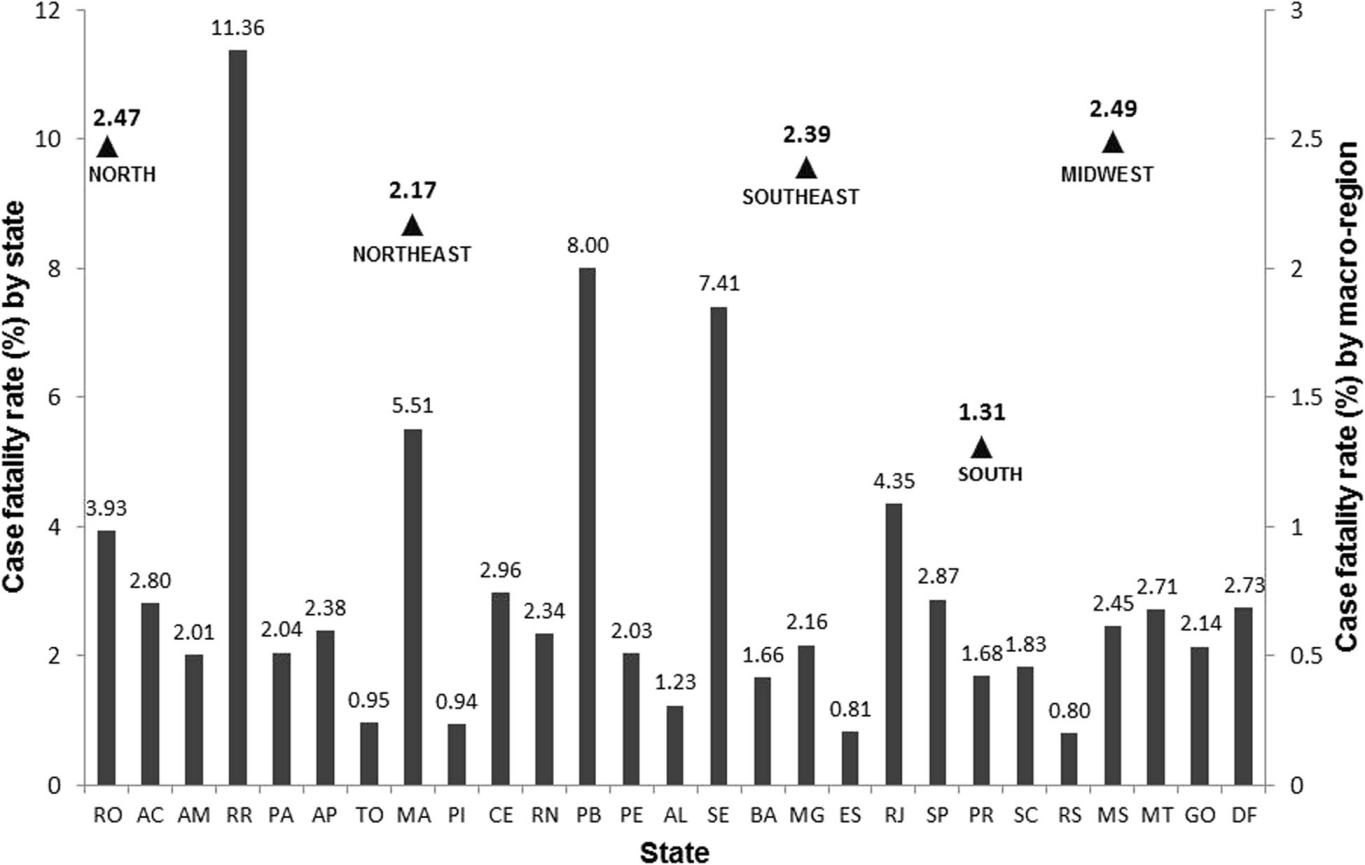
Case-fatality rates of pertussis in Brazil, from 2007 to 2014 in different states and regions. Case fatality rates were calculated as the number of deaths/number of confirmed cases X 100

The overall case fatality rate among confirmed cases was 2.1 % (528 per 24,612). However, it was observed that among children under 3 months of age, mortality was substantially higher, reaching 4.7 % (401/8494). Figure 6 shows the annual distribution of pertussis-associated deaths, the evolution of case fatality rates from 2007 to 2014, and the mortality in specific age groups. A comparison of the case fatality rates in different regions of Brazil demonstrates the lowest rates in the South (1.3 %); Rio Grande do Sul state registered the lowest fatality rate (0.8 %). The Midwest region registered the highest case fatality rate among the macro-regions (2.5 %); however the state with the highest proportion of deaths was Roraima (North Region), which had a fatality rate of 11.4 % (Fig. 7). The complications most commonly reported in the notification forms included pneumonia, encephalitis, dehydration, otitis, and malnutrition. In Table 1, we compare the frequency of these complications reported among the patients who progressed to hospital discharge or death in different age groups. Significantly higher frequencies of pneumonia, encephalitis, dehydration and malnutrition were observed in the group of children who progressed to death.

**Table 1:** Frequency of different complications in fatal and non-fatal pertussis cases in different age groups in Brazil, 2007–2014. The rates displayed are the number of children with the specific complication/total number of children in each category (cured or fatal cases). Comparison of proportions was performed with the chi-square test

		Complications				
Age group		*Pneumonia*	*Encephalopathy*	*Dehydration*	*Otitis*	*Malnutrition*
0–2 months	Cured cases	1237/6787 (18.2 %)	88/6763 (1.3 %)	159/6778 (2.3 %)	44/6748 (0.7 %)	71/6765 (1 %)
	Fatal cases	233/370 (63 %)	24/346 (6.9 %)	34/351 (9.7 %)	2/341 (0.6 %)	16/349 (4.6 %)
	*p*-Value	<0.001	<0.001	<0.001	0.84	<0.001
3–6 months	Cured cases	701/4550 (15.4 %)	49/4534 (1.1 %)	117/4533 (2.6 %)	64/4523 (1.4 %)	50/4526 (1.1 %)
	Fatal cases	57/83 (68.7 %)	9/78 (11.5 %)	10/79 (12.6 %)	2/78 (2.6 %)	4/78 (5.1 %)
	*p*-Value	<0.001	<0.001	<0.001	0.39	0.006
7–15 months	Cured cases	243/1764 (13.8 %)	14/1757 (0.8 %)	44/1758 (2.5 %)	46/1756 (2.6 %)	29/1758 (1.6 %)
	Fatal cases	13/21 (61.9 %)	1/19 (5.2 %)	0	1/20 (5 %)	0
	*p*-Value	<0.001	0.39	-	0.96	-
16 months–4 years	Cured cases	236/2612 (9 %)	15/2610 (0.6 %)	53/2615 (2 %)	51/2607 (2 %)	33/2609 (1.3 %)
	Fatal cases	3/10 (30 %)	0	0	0	2/10 (20 %)
	*p*-Value	0.08	-	-	-	<0.001
5–14 years	Cured cases	115/2557 (4.5 %)	9/2550 (0.3 %)	42/2558 (1.6 %)	64/2555 (2.5 %)	23/2551 (0.9 %)
	Fatal cases	1/4 (25 %)	1/4 (25 %)	0	0	0
	*p*-Value	0.44	<0.001	-	-	-
>15 years	Cured cases	83/2152 (3.9 %)	5/2151 (0.2 %)	18/2154 (0.8 %)	38/2153 (1.8 %)	8/2152 (0.4 %)
	Fatal cases	1/4 (25 %)	0	0	0	1/4 (25 %)
	*p*-Value	0.37	-	-	-	<0.001

### Vaccination status of notified and confirmed pertussis cases and pertussis vaccine coverage

It was noted that 10,600 confirmed cases of pertussis (43 % of the total) occurred in people over the age of 7 months (the age group that should have received a minimum of 3 doses of vaccine against pertussis). Of this group, there was information about the vaccination status of 6948 subjects. These cases were divided into two groups: fully vaccinated (3 doses), with 5687 subjects, and incompletely vaccinated (less than 3 doses), with 1261 individuals. Thus, at least 23.1 % of the confirmed pertussis cases occurred in subjects who had received at least 3 doses of the pertussis vaccine. Within this group, there were 2079 children aged 16 months to 4 years and 1098 infants aged 7–15 months. Thus, it can be considered that 12.9 %, i.e., (2079 + 1098)/24.612 cases of pertussis confirmed in Brazil between 2007 and 2014, occurred in fully immunized infants and children aged 7 months to 4 years. Among these 3177 cases, 911 were confirmed using laboratory criteria; this value corresponds to 3.7 % of confirmed pertussis cases in Brazil between 2007 and 2014. Among the 1261 cases reported in incompletely vaccinated subjects older than 7 months of age, the predominant age group was between 7 and 15 months (*n* = 515).

Table 2 shows that the confirmation rates were significantly lower in children with a complete vaccination schedule (3 or more doses) compared with those with incomplete immunization (2 doses or less), both in the group aged 7 to 15 months and in the group aged 16 months to 4 years. However, beginning in June 2012, which coincided with the replacement of DTwP-Hib by the DTwP-Hib-HBV vaccine, the difference between the confirmation rates among completely and incompletely vaccinated children decreased, primarily in the group aged 16 months to 4 years.

**Table 2:** Pertussis confirmation rates by vaccination status in different periods and age groups, Brazil, 2007–2014

		Confirmation rates		
	Notified cases (N)	Fully vaccinated	Incompletely vaccinated or unvaccinated	*p*-value
Period/Age group				
Jan 2007 – May 2012				
7–15 months	1797	379/1292 (29,3 %)	212/505 (42 %)	<0.001
16–48 months	3319	809/2964 (27.3 %)	132/355 (37.2 %)	<0.001
Period /Age group				
Jun 2012–Dec 2014				
7–15 months	3635	719/2722 (26.4 %)	303/913 (33.2 %)	<0.001
16–48 months	9283	2659/8548 (31.1 %)	261/735 (35.5 %)	0.013

**Fig. 8:**
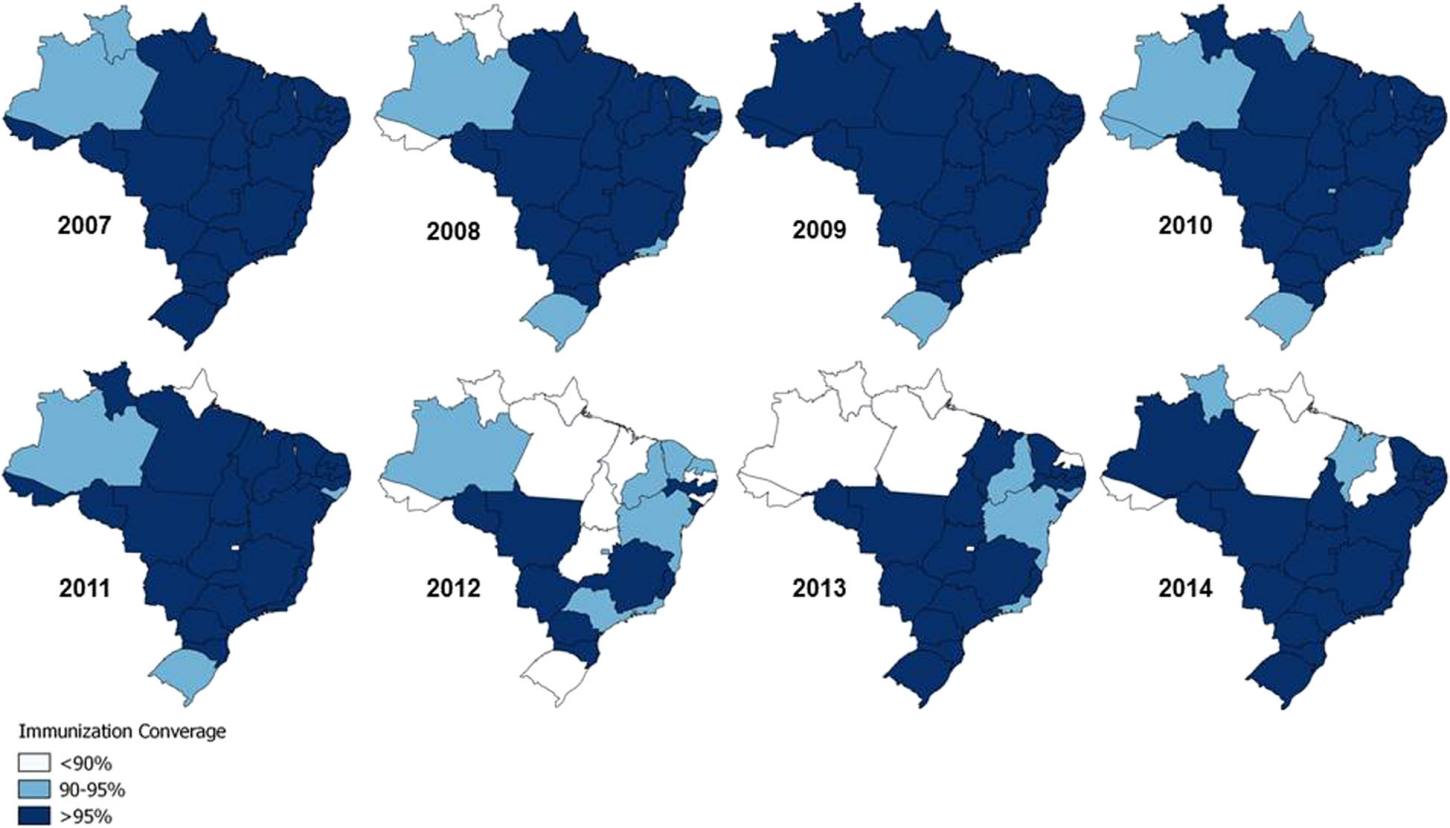
Pertussis vaccine coverage in different Brazilian states by year, from 2007 to 2014. Immunization coverage rates are calculated as the number of children with complete basic scheme in the target age for a particular type of vaccine/number of children in the target age X 100 in different Brazilian states. The maps were made with the QGIS program using study data

**Fig. 9:**
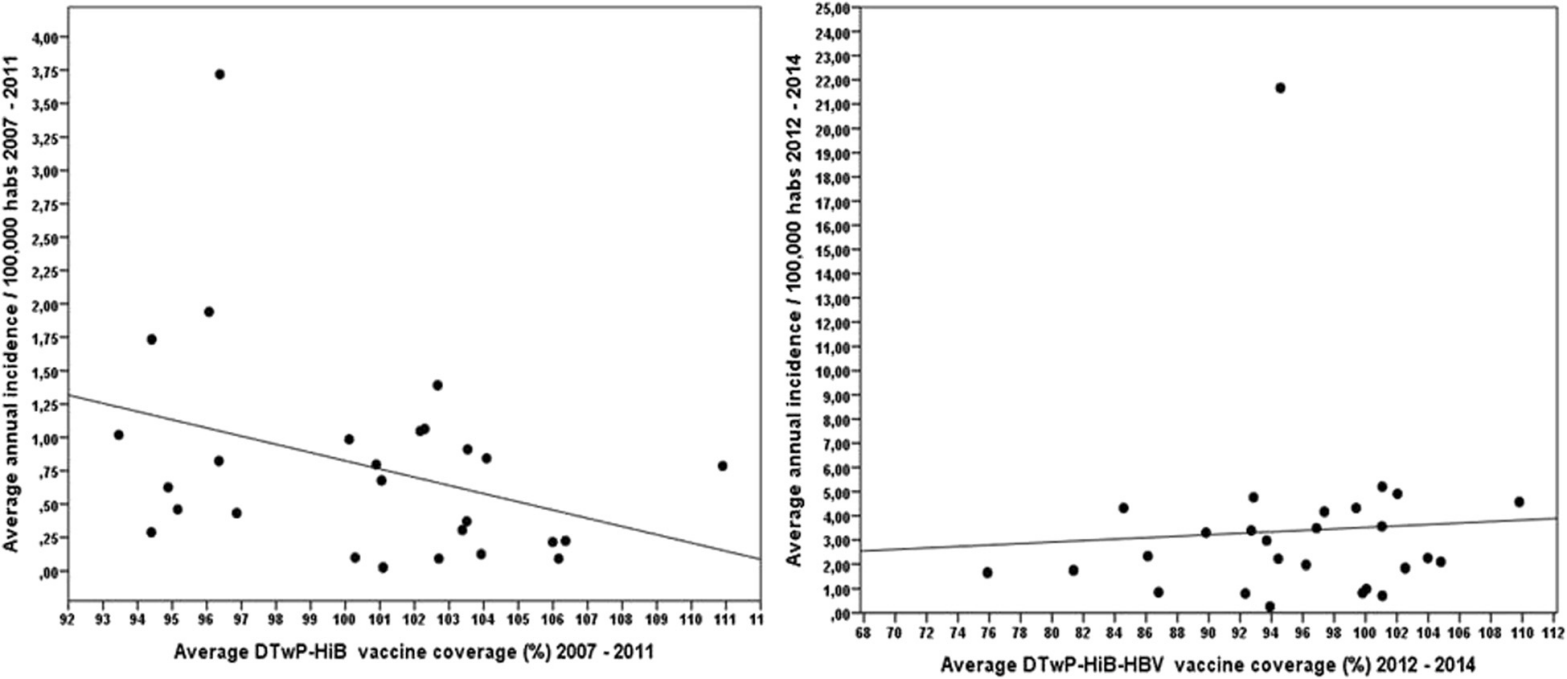
Scatter diagrams showing the correlation between average annual pertussis vaccination coverage (number of children with complete basic scheme in the target age for a particular type of vaccine/number of children in the target age X 100) and average annual pertussis incidence (number of pertussis confirmed [by any criteria] cases/population in each state in a specific year X 100,000 inhabitants) by state in two different periods: 2007–2011 (diphtheria-tetanus- whole pertussis + *H. influenzae* type B vaccine) and 2012–2014 (diphtheria-tetanus- whole pertussis + *H. influenzae* type B + hepatitis B vaccine). From 2007 to 2011, R = 0.358; *p* = 0.067. From 2012 to 2014, R = 0.059; *p* = 0.769

In 2007, two states did not reach 95 % vaccination coverage, and this number increased to six states in 2008. In 2012, 18 states did not achieve 95 % immunization coverage, a number that dropped to ten and six in 2013 and 2014, respectively (Fig. 8). As presented in Fig. 9, during the pre-epidemic period (2007–2011), there was a trend of correlation between pertussis incidence and vaccine coverage (R = 0.358; *p* = 0.067). Nevertheless, from 2012 to 2014, when incidence rates reached epidemic levels, such correlation was not observed (R = 0.059; *p* = 0.769).

## Discussion

A global resurgence of pertussis has been observed beginning in the 1990s and the early 21^st^ century. This study demonstrates a re-emergence of pertussis in Brazil, especially since 2012. In the US, since 1993, the incidence of pertussis has increased, reaching over 25,000 cases per year in 2004 and 2005, 48,277 cases in 2012, and approximately 28,000 cases per year in 2013 and 2014 [15], with some localized outbreaks [2, 16, 17]. In Europe, most countries recorded an increase in the number of cases, especially the Netherlands, UK, Denmark and Norway. Between 2003 and 2007, the overall average annual incidence was 4.1/100,000, rising to 4.9/100,000 in 2009 [18–20]. In some countries, such as the Netherlands, abrupt increases occurred [21]. Data from this study suggest that epidemic levels were reached in Brazil in 2011, increased in 2012, and reached another peak in 2014. However, these values are still below those observed in other countries, such as in Europe, possibly because of underreporting.

In any epidemiological surveillance system, there is concern about underreporting. It has been demonstrated through capture-recapture methods that pertussis is significantly underreported; in the US, it is likely that the actual number of cases is 5–6 times than the reported number. This failure to report cases may be even greater for teenagers and adults because the symptoms are usually mild and may go unnoticed [22–24]. Thus, the actual state of the disease in Brazil may be even more alarming.

Cyclic epidemic peaks of pertussis are observed in many countries. The interval between these peaks varies from one study to another, suggesting that they occur every 2–5 years [2, 6, 18, 25, 26]. Brazilian data show increases in the years 1997, 1998, 2004 and 2005. This study shows increasing incidence from 2011 to 2014; in 2014, it reached the highest number of cases in 30 years [27].

This study shows that pertussis has a clear seasonal pattern in Brazil, with an increase in the number of cases between late spring and summer (in the Southern Hemisphere), specifically in the months of November, December, January and February and especially in South and Southeast regions. Similarly, it has been reported that in the Northern Hemisphere, pertussis occurs most frequently in summer and autumn [18]. Interestingly, in Brazil, other acute respiratory tract infections are more common between the autumn and spring and peak in the winter [28]. These differences in seasonal patterns may be useful for identifying and diagnosing pertussis.

The age distribution of pertussis cases vary between the distinct regions of the world and from one period to another. During an epidemic in the US, the incidence was higher among children under 6 months of age; during the second epidemic, the incidence increased considerably for children aged 14–16 years [2, 17]. Similar variations in age distribution have been reported in Europe [20, 29].

In the present study, we found that 1/3 of the confirmed cases occurred in infants aged 0 to 2 months, an age at which they have not received any dose of a pertussis vaccine. A little over half the cases occurred in children under the age of seven months, i.e., while they were in the process of immunization. During the period of increased incidence (2012–2014), the proportion of confirmed cases in not fully immunized subjects decreased, while an increase in the proportion of cases in older children and adolescents was observed.

Regarding the diagnostic criteria used, most cases were confirmed by clinical and epidemiological criteria, and laboratory confirmation was obtained in a little more than one-third of cases. This is a low proportion; however, it can be explained by the difficulties of obtaining a nasopharyngeal swab in most hospitals and health centres. Nevertheless, laboratory tests are important for confirming pertussis because clinical diagnosis can be difficult [30–32]. In the US, the significant increase in the number of cases may be associated with the introduction of more accurate techniques, such as PCR [16, 33]. In Mexico, there was a substantial increase in pertussis confirmations when PCR testing was implemented [26]. Interestingly, in Italy, the case definition is based on clinical criteria, and laboratory confirmation is not routine [29]. In Spain, the proportion of laboratory confirmation has gradually increased, reaching 69 % [34]. In the US, each confirmed case is linked to a positive laboratory test or is epidemiologically associated with a case confirmed by culture or PCR [35]. Unfortunately, the culture of nasopharyngeal secretions of *B. pertussis,* despite high specificity, presents low sensitivity, and its applicability is subject to logistic limitations. The state of São Paulo, which has used the PCR technique since 2009, had the highest rate of laboratory confirmation of the country. In general, the Southern and Southeastern regions had the highest laboratory confirmation rates in Brazil. Efforts to improve pertussis laboratory diagnosis capacity in Brazil should be undertaken, health professionals involved in patient care in health services should be engaged, and laboratory infrastructure within the Unified Health System should be improved.

The pertussis complications are more prevalent in children under one year of age. In this study, pneumonia was the most commonly observed complication, especially in infants under 3 months of age. In California in 2010, 18.8 % of patients with pertussis had pneumonia. In Russia, the most frequent complications were encephalopathy, followed by bronchitis and pneumonia [17, 36]. A study in the state of Paraná in Brazil also showed that pneumonia was the complication most often associated with death [13]. The overall pertussis case fatality rate in Brazil is higher than that of other countries. In Canada between 1991 and 1997, there was a fatality rate of 0.9 %, less than half the rate observed in Brazil between 2007 and 2014 [37, 38]. Additionally, the absolute number of deaths from pertussis in Brazil is higher than those observed in other countries. In England between 2001 and 2011, there were 48 deaths, the same number of deaths reported in a 6-month period during the epidemic years of the disease in Brazil [39]. In the US in 2013, there were only 13 deaths from pertussis, with a case fatality rate of 0.02 % [40].

A global resurgence of pertussis has been noticed, especially in developed countries. Major hypotheses to explain it include a lower immunogenicity of aP compared with wP vaccines, which would lead to the early weaning of vaccine-induced immunity, the circulation of *B. pertussis* among adolescents and adults and, from these groups, the infection of infants who are not fully immunized. This phenomenon may also be associated with vaccines’ selective pressure on circulating strains, which could cause them to block the antibodies produced by immunization [39].

However, in Brazil, aP vaccines have never been introduced universally and are available only in private vaccination clinics. The proportion of Brazilian children and adults who have been immunized with aP vaccines is not known. Vaccines containing the aP component are available in the NIP only to pregnant women and children who have developed serious side effects after the use of wP vaccines. Thus, the increase in the incidence of pertussis observed in recent years in Brazil should have other possible explanations.

This study reveals that most cases of pertussis reported in Brazil from 2007 to 2014 occurred in subjects older than 6 months of age, with almost 13 % of these cases recorded in fully immunized infants and children aged 7 months to 4 years. Almost 1/3 of these cases were laboratory confirmed. These data point to the possibility of some degree of vaccine escape in the context of the DTwP-Hib-HBV vaccine. We observed that among the reported cases, the proportion of confirmed cases was significantly lower among fully vaccinated children, demonstrating the protective effect of the vaccines, although our study did not aim to evaluate the effectiveness of the vaccines currently being used in Brazil. Nevertheless, the difference in vaccination rates among the confirmed and unconfirmed cases was lower during the period of June 2012 through December 2014 compared with the period of January 2007 through May 2012. This may point to a lower performance of DTwP-Hib-HBV compared with the DTwP-Hib.

Interestingly, the increase in the incidence of pertussis in Brazil coincides with the introduction of the DTwP-Hib-HBV to the NIP in 2012, although an initial increase in the incidence was already evident in 2011, before the DTwP-Hib was replaced. It is also worth noting that during this period, there was a relative reduction in vaccine coverage, which increased the number of states that did not reach 95 % coverage. In the present study, we found that 2 % of the confirmed cases occurred in incompletely vaccinated infants older than 6 months of age. During the pre-epidemic period there was a trend for states with lower vaccination coverage present the highest rates of incidence of pertussis. This pattern was not observed in the 2012–2014 period.

In 2013, Brazil introduced the DaPT for pregnant women as a measure to contain the resurgence of pertussis. Brazil’s main challenges in facing pertussis resurgence will be to offer the highest quality medical attention to reduce mortality, to improve the infrastructure for laboratory diagnoses, and to maintain high vaccination coverage. Additional studies to assess the effectiveness of the current vaccination schedule, including basic research on the genetics and evolution of circulating *B. pertussis* strains, are also needed.

## Conclusions

There has been a significant increase in the incidence of pertussis in Brazil, especially since 2012. A little over half of the cases occurred in infants younger than 7 months of age. Some of the confirmed cases may be associated with primary vaccine failure, as some have occurred in vaccinated children aged 7 months to 4 years. Laboratory confirmation rates are less than ideal and have large regional differences. Fatality rates are high compared with those described in the literature; they are significantly higher in infants and are strongly associated with such complications as pneumonia, malnutrition and dehydration. The increase in incidence coincides with the introduction of the pentavalent vaccine and with a relative reduction in vaccination coverage in several states. Brazil’s challenges in facing rising pertussis rates will be to offer the highest quality medical care, improve the infrastructure for laboratory diagnostics and maintain high vaccination coverage.

## Consent

Consent was not considered necessary by the Research Ethics Committee of the Instituto Oswaldo Cruz,as it is a study with secondary data from reporting and surveillance records.

## Abbreviations

DTP: Diphtheria-tetanus-pertussis vaccine
wP: whole pertussis vaccine
aP: acelular pertussis vaccine
NIP: National Immunization Program
DTwP: Diphtheria-tetanus- whole pertussis vaccine
DTwP-Hib: DTwP + *H. influenzae* type B vaccine
DTwP-HiB-HBV: DTwP + *H. influenzae* type B + hepatitis B
DTaP: Diphtheria-tetanus- acelular pertussis vaccine
DataSUS: Department of Informatics of the Unified Health System
SINAN: Information System for Notifiable Diseases
PCR: Polymerase chain reaction
LACEN: Central Laboratory of Public Health
QGIS: Quantum Geographic Information System
